# TSC22D3-Mediated Quiescence Preservation Boosts HSC Engraftment in Xenografts

**DOI:** 10.3390/biomedicines14071424

**Published:** 2026-06-24

**Authors:** Xiaopeng Hu, Tian Zhang, Guangjin Pan, Xingkui Xue

**Affiliations:** 1 Medical Research Center, People’s Hospital of Longhua, Shenzhen 518000, China; xiaopenghu661@outlook.com (X.H.); baicao27@163.com (T.Z.); 2 CAS Key Laboratory of Regenerative Biology, Joint School of Life Sciences, Guangzhou Institutes of Biomedicine and Health, Chinese Academy of Sciences, Guangzhou Medical University, Guangzhou 510530, China; 3 Guangdong Provincial Key Laboratory of Stem Cell and Regenerative Medicine, South China Institute for Stem Cell Biology and Regenerative Medicine, Guangzhou Institutes of Biomedicine and Health, Chinese Academy of Sciences, Guangzhou 510530, China

**Keywords:** hematopoietic stem cells (HSCs), TSC22D3, quiescence, hematopoietic reconstitution

## Abstract

**Background**: Hematopoietic stem cell (HSC) ex vivo culture causes severe loss of repopulation and regenerative capacity without compromising multilineage differentiation, which greatly limits the efficacy of HSC transplantation. The molecular mechanisms underlying culture-triggered HSC dysfunction remain poorly understood. **Methods**: Human CD34^+^ HSCs were cultured ex vivo for 96 h to establish a culture-induced HSC dysfunction model. Single-cell RNA sequencing was applied to screen key regulatory genes. TSC22D3 function was verified via overexpression assays, and immunodeficient mice were used to assess HSC engraftment. Transcriptomic profiling were performed to explore downstream molecular mechanisms. **Results**: Ex vivo culture induced G0 quiescence exit, elevated early apoptosis and impaired in vivo repopulation in human CD34^+^ HSCs. TSC22D3 was highly enriched in freshly isolated quiescent HSCs and gradually downregulated during culture. TSC22D3 overexpression restored HSC G0 arrest and improved hematopoietic engraftment in mice. Mechanically, TSC22D3 upregulated HSC self-renewal genes, suppressed cell cycle-related genes (CDK2/4), and activated the P53-P21-P27 pathway. **Conclusions**: This study demonstrates that TSC22D3 preserves HSC function during ex vivo culture by maintaining stem cell quiescence and restricting excessive proliferation. These findings reveal a novel transcriptional mechanism regulating HSC homeostasis and provide a promising target for improving functional HSC ex vivo expansion for clinical transplantation.

## 1. Introduction

Hematopoietic stem cell (HSC) transplantation serves as a curative treatment for a wide range of hematological malignancies and inherited genetic disorders [1,2,3]. Nevertheless, its widespread clinical use is largely restricted by the limited availability of functional HSCs, as well as the rapid loss of repopulating capacity during ex vivo expansion. Most routine transplantations adopt freshly isolated, non-expanded HSC products, but inadequate cell doses often lead to delayed hematopoietic reconstitution and poor engraftment. For example, umbilical cord blood, a commonly used graft source, contains inherently low numbers of stem cells and thus fails to achieve rapid engraftment in adult recipients. Likewise, HSCs isolated from patients with aplastic anemia are also quantitatively insufficient, which makes ex vivo expansion an indispensable approach to meet clinical transplant requirements. Unfortunately, although cultured HSCs retain multilineage differentiation potential, they tend to exit the quiescent state [4,5], undergo abnormal proliferation and eventually become functionally exhausted. The molecular mechanisms underlying this phenomenon remain largely elusive. As a core characteristic of HSCs, quiescence (G0 phase) sustains their long-term self-renewal and hematopoietic reconstitution abilities [6,7]. Accumulating evidence has demonstrated that transcriptional and epigenetic modulators are central to maintaining HSC quiescence [8,9,10,11]. Even so, the key transcription factors that protect HSC function during in vitro expansion have not been fully identified.

*TSC22D3* (also known as *L-GLZ*) is a transcription factor previously implicated in tumor suppression [12,13], where it inhibits cell proliferation by activating the P53-P21 pathway [12,14,15]. However, its physiological function in normal hematopoiesis, particularly in the context of HSC homeostasis, has not been explored. Here, we sought to identify the molecular drivers of culture-induced HSC dysfunction and investigate the role of TSC22D3 in regulating HSC quiescence and repopulation capacity.

Using single-cell RNA sequencing (scRNA-seq) and functional assays, we demonstrate that 96 h of in vitro culture drives HSCs out of quiescence, triggers early apoptosis, and abrogates in vivo reconstitution—phenotypes that correlate with the progressive downregulation of TSC22D3. Gain-of-function experiments reveal that TSC22D3 overexpression restores G0 arrest, enhances hematopoietic reconstitution in immunodeficient mice, and modulates a transcriptional program that upregulates established HSC self-renewal genes (e.g., *MLLT3*, *TXNIP*, *KLF6*, *ICAM1*) while repressing cell cycle and metabolic drivers (e.g., *CDK4*, *CDK2*, *CDC6*) [16,17,18]. Mechanistically, *TSC22D3* activates the *P53*-*P21*-*P27* axis, a well-characterized pathway that enforces HSC quiescence and prevents exhaustion, and our working model suggests that *TSC22D3* acts via a cytoplasmic partner to translocate to the nucleus and fine-tune transcriptional networks that balance quiescence and proliferation.

These findings identify *TSC22D3* as a pivotal regulator of HSC homeostasis and provide a novel strategy to preserve HSC function during ex vivo expansion, with potential implications for improving the efficacy of HSC-based clinical therapies.

## 2. Materials and Methods

### 2.1. Lentiviral Vector Construction and Production for TSC22D3 Overexpression

The full-length cDNA of human TSC22D3 was amplified from human hematopoietic stem and progenitor cells (HSPCs) and cloned into the TetO-FUW lentiviral vector using recombinant DNA technology. The TSC22D3 cDNA was placed under the control of the Tet-on promoter, fused in-frame with an eGFP sequence at the C-terminus, and linked to a puromycin resistance gene via a P2A or T2A self-cleaving peptide sequence. All primers used for cloning are listed in Table 1.

Lentivirus was produced in 293T cells by co-transfecting the TetO-FUW-TSC22D3-eGFP vector with three packaging plasmids (pRSV-REV, Addgene #12253; pMDLg/pRRE, Addgene #12251; pMD2.G, Addgene #12259). Viral supernatants were collected at 36 h and 60 h post-transfection, filtered through a 0.45 μm filter (Millipore, Billerica, MA, USA, catalog No. SLFH05010) to remove cell debris, and concentrated by ultracentrifugation at 50,000× *g* for 2.5 h at 4 °C. Viral titers were determined using the Lenti-X™ qRT-PCR Titration Kit (Clontech Laboratories, Mountain View, CA, USA, catalog No. 631235) following the manufacturer’s instructions.

### 2.2. Lentiviral Transduction of Human HSPCs

CD34+ HSPCs were isolated from mobilized peripheral blood of healthy volunteers using MACS (Miltenyi Biotec, Bergisch Gladbach, Germany, catalog No. 130-046-703), with approval from the Institutional Review Board (IRB) of Longhua District People’s Hospital of Shenzhen. Isolated CD34+ HSPCs were pre-stimulated for 24 h in SCGM medium (CellGenix, Freiburg im Breisgau, Germany, catalog No. 208020500) supplemented with 100 ng/mL human SCF (PeproTech, Rocky Hill, NJ, USA, catalog No. 300-07), 100 ng/mL human TPO (Sino Biological, Beijing, China, catalog No. 10381-H08H), 100 ng/mL human FLT3-L (Sino Biological, Beijing, China, catalog No. 10315-H07B), and penicillin–streptomycin (HyClone, Logan, UT, USA, catalog No. SV30010).

Pre-stimulated HSPCs were then seeded into wells pre-coated with 5 μg/cm^2^ fibronectin (Sigma-Aldrich, St. Louis, MO, USA, catalog No. F2006) for 6 h. The culture medium was supplemented with 5 μg/mL protamine sulfate (Sigma-Aldrich, St. Louis, MO, USA, catalog No. P3369), 10 μM prostaglandin E2 (Cayman Chemical, Ann Arbor, MI, USA, catalog No. 363-24-6), and 8 nM Cyclosporin H (Cayman Chemical, Ann Arbor, MI, USA, catalog No. 17182). Lentivirus encoding TSC22D3-eGFP and rtTA was added at a multiplicity of infection (MOI) of 100. After 24 h of transduction, cells were cultured in SCGM medium containing 20 μg/mL doxycycline (Sigma-Aldrich, St. Louis, MO, USA, catalog No. D9891) to induce TSC22D3 expression. eGFP-positive (eGFP+) cells were sorted by flow cytometry 48 h later and used for subsequent assays.

### 2.3. Assessment of Transduction Efficiency

Transduction efficiency of TSC22D3-eGFP in HSPCs was directly evaluated by flow cytometry (BD Biosciences, San Jose, CA, USA, FACSCanto II) to determine the percentage of eGFP+ cells [19], with eGFP-only transduced HSPCs as the control group.

### 2.4. Cell Cycle Analysis of TSC22D3-Overexpressing HSPCs

Cell cycle distribution was analyzed to assess the quiescent state of HSPCs overexpressing TSC22D3 or eGFP (control). Cells were fixed with Fixation Buffer (BD Biosciences, San Jose, CA, USA, catalog No. 554655) and permeabilized with Permeabilization Solution (BD Biosciences, San Jose, CA, USA, catalog No. 554715). Staining was performed using APC-conjugated Ki67 (Miltenyi Biotec, Bergisch Gladbach, Germany, catalog No. 130-100-330) and DAPI (Thermo Fisher Scientific, Waltham, MA, USA, catalog No. 62248) to distinguish G0 (Ki67^−^/DAPI^+^) from cycling (Ki67+) cells. Samples were analyzed by flow cytometry, and the percentage of cells in the G0 phase was quantified.

### 2.5. Hematopoietic Reconstitution Assay in Immunodeficient Mice

For in vivo transplantation assays, a total of 20 female B-NDG mice (5–8 weeks old) were randomly divided into two experimental groups: control group (eGFP only, *n* = 10) and TSC22D3 overexpression group (TSC22D3-eGFP, *n* = 10). Each mouse received an intravenous injection of approximately 250,000 sorted eGFP-positive HSPCs after sublethal irradiation. All in vitro experiments were independently repeated at least three times with three biological replicates. Peripheral blood was collected at multiple time points post-transplantation, and red blood cells were lysed using ACK Lysis Buffer (Leagene Biotechnology, Jinan, Shandong, China, catalog No. CS0001). Human hematopoietic reconstitution capacity was evaluated by flow cytometry to detect the percentage of human CD45+ (hCD45+) cells in mouse peripheral blood 4 weeks after transplantation. All antibodies used for flow cytometry are listed in Table 2.

### 2.6. Single Cell RNA-Seq

Lin-CD34+CD38−CD45RA−CD90+ HSPCs isolated from mobilized peripheral blood and cultured for 0 h, 48 h, 72 h and 96 h were sorted into 96-well plates by a capillary pipette. Cell lysis and cDNA synthesis were conducted as previously described [20]. Genomic DNA libraries were constructed with 5 ng of cDNA using the TruePrep™ DNA Library Prep Kit V2 for Illumina^®^ (Vazyme Biotech Co., Ltd., Nanjing, China, Cat# TD503), followed by sequencing on the NextSeq 500 platform (Illumina Inc., San Diego, CA, USA).

scRNA-seq data analysis was performed with the Scanpy Python toolkit v1.12 (https://scanpy.readthedocs.io). Low-quality cells with fewer than 200 expressed genes per cell and genes expressed in fewer than 3 cells were filtered out. The expression matrix was then normalized by total counts per cell and scaled to transcripts per 10,000 (TP10K) for intercellular comparison, followed by log(X + 1) transformation (natural logarithm). Highly variable genes (HVGs) were identified using the highly_variable_genes function with the parameters min_mean = 0.0125, max_mean = 10 and min_disp = 0.5, and the dataset was further scaled to zero mean and unit variance. For dimensionality reduction, 100 principal components (PCs) were calculated, and the top 50 PCs were retained for subsequent cell clustering and UMAP projection based on PCA variance ratio.

Pseudotime analysis was implemented with Monocle to construct single-cell developmental trajectories and calculate pseudotime values for cells at different culture time points. HVGs with a q-value < 0.01 across all time points were selected, discriminative dimensionality reduction (DDRTree) was performed for dimensionality reduction and trajectory construction, and all cells were ordered along the generated trajectory according to their pseudotime values.

Raw scRNA-seq data have been deposited in the Gene Expression Omnibus (GEO) database under the accession code GSE176318 (https://www.ncbi.nlm.nih.gov/geo/query/acc.cgi?acc=GSE176318, accessed on 15 June 2026).

### 2.7. qPCR Analysis

First, 293T cells were transfected with lentiviral vectors encoding TSC22D3 or empty vector (control). Total RNA was extracted, and cDNA was synthesized. Quantitative real-time PCR (qPCR) was performed to detect the mRNA expression levels of p21 and p27. Relative gene expression was calculated using the 2^−ΔΔCt^ method, with GAPDH as the internal reference [21].

### 2.8. Western Blot

293T cells transfected with TSC22D3 or control vectors were lysed in RIPA buffer containing protease inhibitors. Protein lysates were separated by SDS-PAGE and transferred to PVDF membranes. Membranes were probed with primary antibodies against P53, P21, P27, and GAPDH (loading control), followed by horseradish peroxidase (HRP)-conjugated secondary antibodies. Protein bands were visualized using an ECL detection system, and densitometric analysis was performed to quantify protein expression levels. All antibodies used for WB are listed in Table 2.

### 2.9. Statistical Analysis

Statistical analysis was performed using GraphPad Prism v8.0.2 software. Data are presented as mean ± standard deviation (SD). Differences between groups were analyzed using an unpaired two-tailed Student *t*-test. Statistical significance was defined as * *p* < 0.05 and ** *p* < 0.01. No samples were excluded from the analysis.

## 3. Results

### 3.1. In Vitro Culture Compromises the Repopulation Capacity of HSCs Without Altering Differentiation Potential

**Figure 1:**
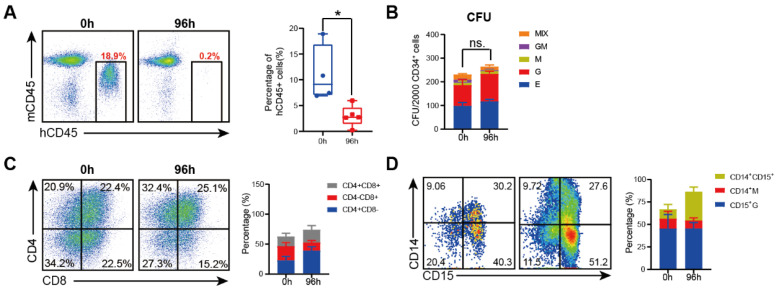
Changes in hematopoietic stem cells (HSCs) after in vitro culture. (**A**) Percentage of human-derived cells in peripheral blood after transplantation of HSCs cultured for 0 h and 96 h into immunodeficient mice analyzed by flow cytometry. (**B**) Colony formation of HSCs cultured for 0 h and 96 h. (**C**) T cell differentiation capacity of HSCs in vitro. (**D**) Comparison of monocyte differentiation of HSCs after in vitro culture. Statistical significance was determined by an unpaired two-tailed Student’s *t*-test. Data are presented as mean ± SD (*n* = 3). Differences were considered significant at * *p* < 0.05; ns., not significant.

CD34^+^ hematopoietic stem and progenitor cells (HSPCs) were isolated from mobilized peripheral blood samples collected from three healthy volunteers, cultured in vitro for 96 h, and then transplanted into immunodeficient mice. The hematopoietic reconstitution ability of cultured HSCs was significantly reduced compared with freshly isolated HSCs (Figure 1A). However, cultured HSCs remained capable of forming erythroid, myeloid, macrophage and mixed colonies, with no significant difference in colony-forming capacity relative to uncultured HSCs (Figure 1B). In vitro differentiation assays showed that both freshly isolated and cultured HSCs differentiated into CD4^+^/CD8^+^ T lymphocytes and CD15^+^/CD14^+^ granulocytes/monocytes (Figure 1C,D). These results indicate that in vitro culture preserves the differentiation capacity of HSCs but severely impairs their in vivo hematopoietic repopulation potential.

### 3.2. In Vitro Culture Induces HSC Exit from Quiescence

**Figure 2:**
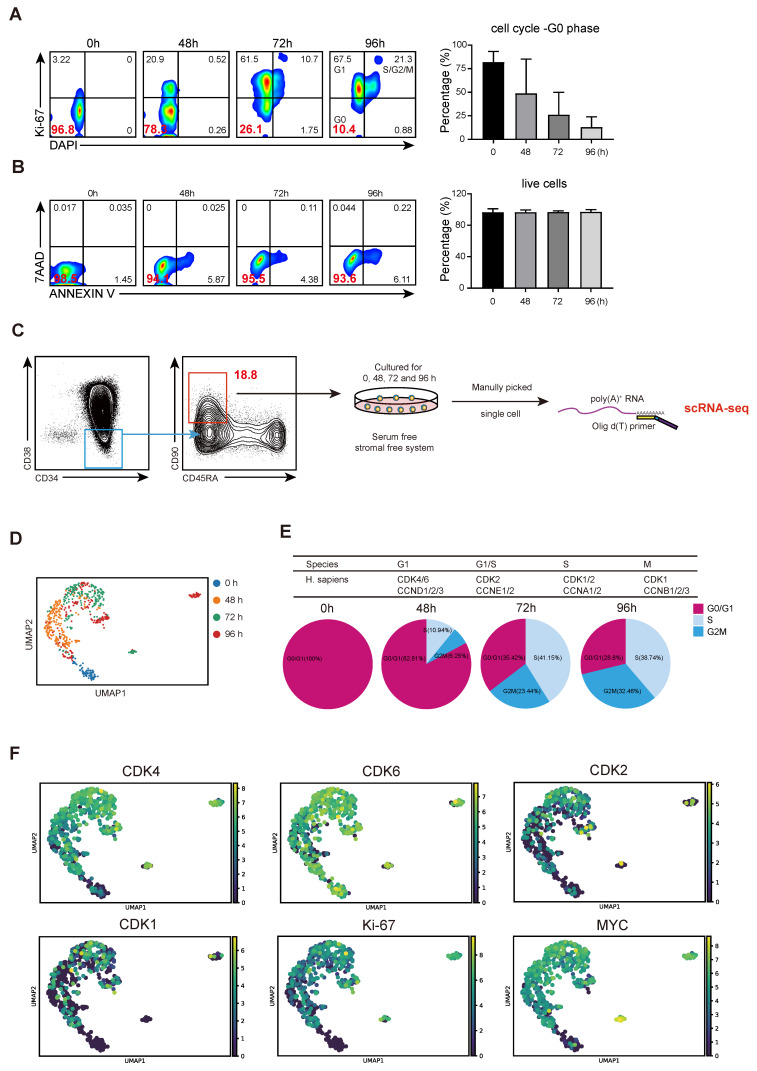
In vitro culture induces exit of HSCs from quiescence. (**A**) Cell cycle distribution of HSCs analyzed by flow cytometry. (**B**) Apoptosis of HSCs determined by flow cytometry. (**C**) Schematic diagram of the single-cell RNA-seq analysis. (**D**) UMAP plot showing the distribution of single cells at different time points. (**E**) Cell cycle status of HSCs at different time points analyzed by RNA-seq. (**F**) Expression of cell cycle-related genes examined by qPCR. Quantitative data in (**A**,**B**) are presented as mean ± SD from three independent biological replicates (*n* = 3).

Cell cycle analysis demonstrated that in vitro culture drove HSCs into active proliferation and out of quiescence (Figure 2A). Apoptosis assays revealed that the proportion of early apoptotic HSCs increased to approximately 5% after in vitro culture (Figure 2B). Single-cell RNA sequencing (scRNA-seq) and UMAP analysis were performed on CD34^+^CD38^+^Lin^+^CD90^+^CD45RA^+^HSCs at 0, 48, 72 and 96 h of in vitro culture (Figure 2C,D). Transcriptomic analysis confirmed the progressive loss of G0 quiescence in cultured HSCs, accompanied by gradually elevated expression of cell cycle-related genes including CDK4 and Ki-67 (Figure 2E,F). These data suggest that the impaired repopulation capacity of HSCs after in vitro culture is closely associated with exit from quiescence.

### 3.3. TSC22D3 Is Highly Expressed in Quiescent HSCs and Downregulated During In Vitro Culture

**Figure 3:**
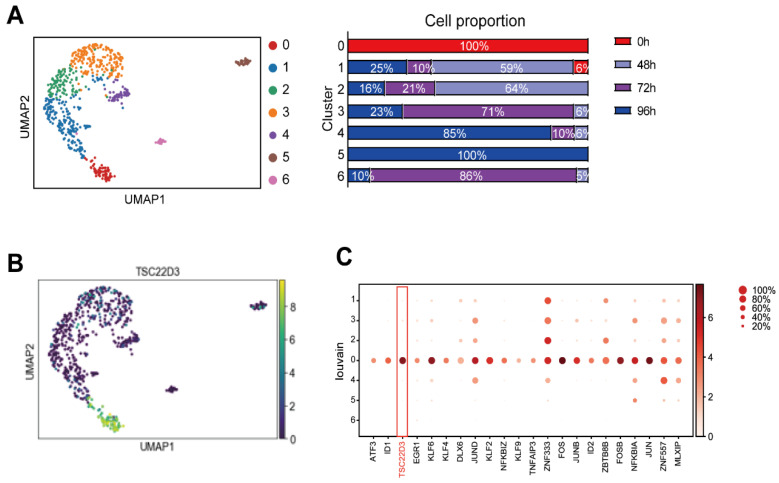
Transcriptomic difference analysis of HSCs during in vitro culture. (**A**) UMAP analysis showing the proportion of cells at each time point of in vitro culture in the cell population. All percentage values are rounded to whole integers. (**B**) Expression of transcription factor TSC22D3 in different cell populations. (**C**) Expression analysis of the top 22 genes highly expressed in uncultured HSCs among each cell population.

scRNA-seq analysis clustered HSCs at different culture time points into six distinct cell populations (Figure 3A,B). Cluster 0 showed markedly elevated expression of transcription factors known to regulate HSC quiescence, including KLF2 and NFκB1 (Figure 3C). TSC22D3 was highly abundant in freshly isolated (0 h) HSCs and significantly downregulated during in vitro culture, implying that TSC22D3 may function as a key regulator in maintaining HSC quiescence and repopulation capacity.

### 3.4. TSC22D3 Overexpression Sustains HSC Quiescence and Enhances Hematopoietic Reconstitution

**Figure 4:**
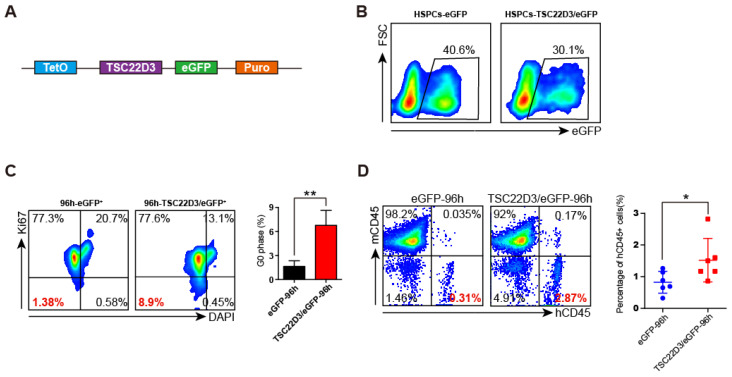
Transcription factor TSC22D3 contributes to maintaining the quiescent state and hematopoietic reconstitution capacity of HSCs. (**A**) Schematic diagram of the vector construction strategy. (**B**) Transfection efficiency of the overexpression vector detected by flow cytometry. (**C**) Cell cycle distribution of HSCs overexpressing TSC22D3 or control eGFP analyzed by flow cytometry. (**D**) Hematopoietic reconstitution capacity of HSCs overexpressing eGFP or TSC22D3/eGFP in immunodeficient mice, as determined by the percentage of human CD45+ cells in peripheral blood. Statistical significance was determined by an unpaired two-tailed Student’s *t*-test. Data are presented as mean ± SD (*n* = 3). Differences were considered significant at ** *p* < 0.01 and * *p* < 0.05.

An inducible TSC22D3 overexpression lentiviral vector was constructed and transduced into in vitro-cultured HSCs (Figure 4A,B). Overexpression of TSC22D3 significantly increased the percentage of G0-phase HSCs compared with the control group (Figure 4C), confirming that TSC22D3 promotes HSC quiescence. Furthermore, HSCs overexpressing TSC22D3 exhibited stronger in vivo hematopoietic reconstitution capacity in immunodeficient mice than control HSCs (Figure 4D).

### 3.5. TSC22D3 Maintains HSC Quiescence and Function via Multiple Biological Processes

**Figure 5:**
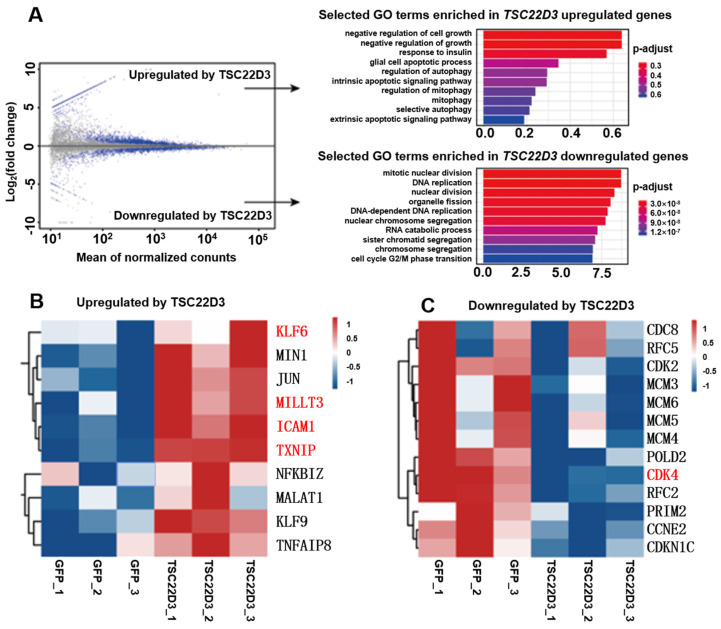
Overexpression of TSC22D3 promotes the maintenance of HSC quiescence via multiple biological processes. (**A**) Gene Ontology (GO) enrichment analysis of pathways associated with upregulated and downregulated genes upon TSC22D3 overexpression in HSCs. (**B**,**C**) Heatmaps showing the expression profiles of significantly upregulated and downregulated genes in TSC22D3-overexpressing HSCs relative to control cells.

Bulk RNA-seq was performed to explore the molecular mechanisms by which TSC22D3 regulates HSC homeostasis. Differential gene analysis showed that TSC22D3 overexpression activated signaling pathways related to HSC function including autophagy and insulin response, while inhibiting pathways associated with DNA replication and cell cycle progression (Figure 5A). At the gene level, TSC22D3 upregulated the expression of established HSC self-renewal regulators, including KLF6, MLLT3, ICAM1 and TXNIP, and downregulated cell cycle-related genes such as CDK4 and CDK2 (Figure 5B,C). These findings suggest that TSC22D3 maintains the quiescence and repopulation capacity of HSCs during in vitro culture through modulating multiple biological processes.

### 3.6. TSC22D3 Overexpression Upregulates P53 Signaling in 293T Cells

**Figure 6:**
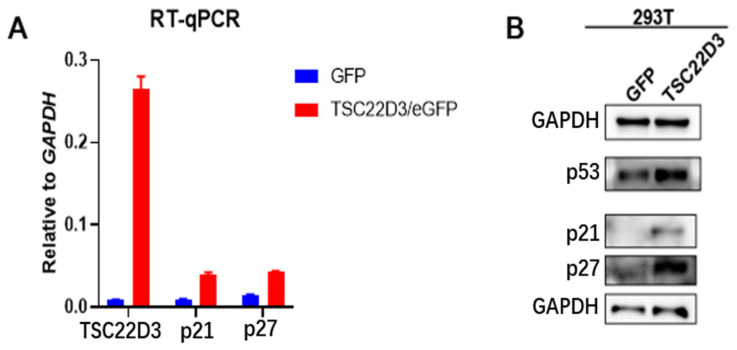
TSC22D3 overexpression increases P53 protein level in 293T cells. (**A**) qPCR analysis showing that overexpression of TSC22D3 promotes the mRNA expression of p21 and p27 in 293T cells. (**B**) Western blot analysis revealing that overexpression of TSC22D3 upregulates the protein expression of P53, P21 and P27 in 293T cells. Quantitative data in panel A are presented as mean ± SD from three independent biological replicates (*n* = 3).

Previous studies reported that TSC22D3 (L-GLZ) inhibits tumor growth by enhancing the expression of p53 and p21, and loss of p21 accelerates HSC cycling and exhaustion. To verify this regulatory mechanism in our system, TSC22D3 was overexpressed in 293T cells. qPCR and Western blot analyses confirmed that TSC22D3 overexpression significantly increased the mRNA and protein levels of P53, P21 and P27, all of which are critical cell cycle regulators (Figure 6A,B). Further validation in primary HSCs and exploration of TSC22D3-interacting proteins via co-immunoprecipitation will be performed to clarify the detailed regulatory mechanism.

## 4. Discussion

Hematopoietic stem cell (HSC) ex vivo expansion remains a critical unmet need for clinical transplantation [22,23], as cultured HSCs rapidly lose repopulation capacity despite preserved differentiation potential [23,24]. Here, we identify TSC22D3 as a pivotal transcription factor that safeguards HSC quiescence and functional integrity during in vitro culture, addressing a key gap in our understanding of HSC extrinsic dysfunction.

Our data confirm that 96 h of in vitro culture drives HSCs out of G0 quiescence, triggers early apoptosis, and abrogates in vivo reconstitution—phenotypes uncoupled from multilineage differentiation capacity. Single-cell transcriptomics further pinpointed TSC22D3 as a quiescence-enriched factor whose downregulation correlates with culture-induced functional decline. Gain-of-function experiments validated this causal link: TSC22D3 overexpression restored G0 arrest and enhanced hematopoietic reconstitution in immunodeficient mice, a finding with direct translational implications.

Clinically, reliable ex vivo HSC expansion has long been pursued to resolve the common challenge of insufficient cell doses in human HSC transplantation. Currently, many clinical transplant procedures rely on limited numbers of primary HSCs, which frequently lead to delayed hematopoietic reconstitution and increased risks of transplant failure. Although multiple cytokine-based culture systems have been developed for HSC expansion in recent years, most protocols fail to sustain long-term stem cell activity, greatly restricting their large-scale clinical application. Therefore, strategies to maintain HSC stemness during culture are urgently required to promote the wide use of ex vivo expansion technologies in human patients.

**Figure 7:**
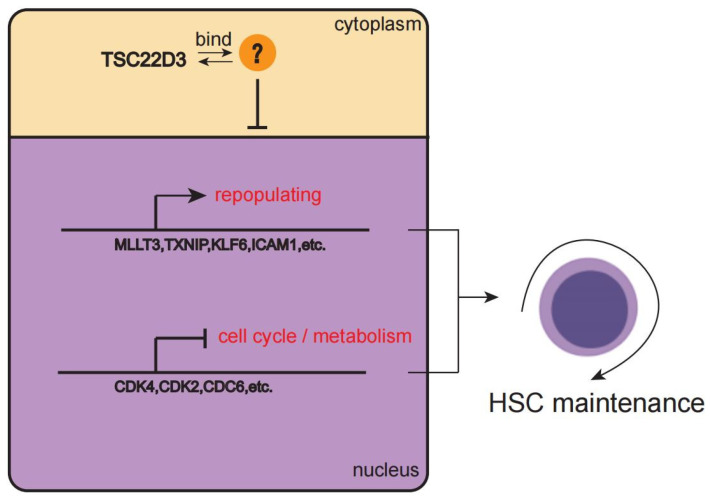
Schematic model illustrating the mechanism by which TSC22D3 maintains hematopoietic stem cell (HSC) homeostasis.

TSC22D3 is a conserved leucine zipper-containing protein belonging to the TSC22 family, which functions as both a transcriptional regulator and an adaptor protein. Previous studies have shown that TSC22D3 can shuttle between the cytoplasm and nucleus, interacting with various partners to regulate its localization and stability. In the nucleus, it acts as a co-factor to modulate the expression of genes involved in cell cycle arrest, apoptosis, and stem cell maintenance. While its detailed binding partners in human HSCs remain to be identified, our working model proposes that TSC22D3 may interact with an unknown cytoplasmic partner to translocate into the nucleus, thereby regulating HSC quiescence and function. Mechanistically, TSC22D3 orchestrates a transcriptional program that reinforces HSC identity—upregulating self-renewal genes (MLLT3, TXNIP, KLF6) and repressing cell cycle drivers (CDK4, CDK2). Concordant with prior reports linking TSC22D3 to tumor suppression, our 293T cell assays demonstrate its ability to upregulate the P53-P21-P27 axis, a well-characterized pathway that enforces HSC quiescence and prevents exhaustion. Collectively, these results support our proposed working model (Figure 7), wherein TSC22D3 likely acts via a cytoplasmic partner to translocate to the nucleus, fine-tuning transcriptional networks that balance quiescence and proliferation.

Notably, this study is limited to in vitro culture and xenograft models; future work will validate TSC22D3’s role in primary human HSCs and preclinical syngeneic models. Identifying the unknown TSC22D3-interacting protein will also be critical to unraveling its full regulatory mechanism. Ultimately, our findings position TSC22D3 as a promising target to optimize ex vivo HSC expansion protocols, potentially expanding the availability of functional HSCs for clinical transplantation.

## Figures and Tables

**Table 1:** Primers.

Primer Name	Primer Sequence (5′-3′)
FUW-TSC22D3-EcoR1-F	CGGAATTCATGGCCCAGTCCAAGCTCGATTGC
FUW-TSC22D3-3×flag Hpa1-R	CGCGGATCCCTTGTCATCGTCATCCTTGTAGTCGATGTCATGATCTTTATAATCACCGTCATGGTCTTTGTAGTCCACCGCAGAACCACCAGGGGC
P21-F	TGTCCGTCAGAACCCATGC
P21-R	AAAGTCGAAGTTCCATCGCTC
P27-F	AACGTGCGAGTGTCTAACGG
P27-R	CCCTCTAGGGGTTTGTGATTCT
GAPDH-F	GTGGACCTGACCTGCCGTCT
GAPDH-R	GTGGACCTGACCTGCCGTCT
CDK4-promoter-F	CAGTCCAAGCTCGATTGCC
CDK4-promoter-R	CTGCCGAAAGTTGCTCACT

All primers used for cloning.

**Table 2:** Antibodies.

Antibodies	Vendor	Cat#	Dilution
PE/Cyanine7 anti-mouse CD45	BioLegend, San Diego, CA, USA	103114	1:100
Anti-human CD34-PerCP-Cy5.5	BD Biosciences, San Jose, CA, USA	347203	1:100
APC/Cyanine7 anti-human CD45RA	BioLegend, San Diego, CA, USA	304127	1:100
CD34 MicroBead Kit, human for 10 × 10	Miltenyi Biotec, Bergisch Gladbach, Germany	130-046-703	
PE/Cyanine7 anti-human CD38	BioLegend, San Diego, CA, USA	303516	1:100
Alexa Fluor^®^ 700 anti-human CD38	BioLegend, San Diego, CA, USA	356623	1:100
PE anti-human CD34	BD Biosciences, San Jose, CA, USA	555822	1:100
Rabbit anti-human HBB antibody	Sigma-Aldrich, St. Louis, MO, USA	HPA043234	1:100
APC anti-Human CD45	BD Biosciences, San Jose, CA, USA	555485	1:100
Human hematopoietic lineage APC cocktail	Thermo Fisher Scientific, Waltham, MA, USA	22-7776-72	1:100
Human hematopoietic lineage FITC cocktail	Thermo Fisher Scientific, Waltham, MA, USA	22-7778-72	1:100
PE/Cyanine7 anti-human CD45	BioLegend, San Diego, CA, USA	304016	1:100
PE/Cyanine7 anti-human CD4	BD BD Biosciences, San Jose, CA, USA	560909	1:100
APC/Cyanine7 anti-human CD8a	BioLegend, San Diego, CA, USA	300926	1:100
DAPI	Beyotime, Shanghai, China	C1005	1:500
7AAD	BD Biosciences, San Jose, CA, USA	559925	1:200
APC anti Ki67	Miltenyi Biotec, Bergisch Gladbach, Germany	130-100-330	1:100
PE/Cyanine7 anti-human Ki67	BioLegend, San Diego, CA, USA	350525	1:100
Alexa Fluor 568 goat anti-mouse	Thermo Fisher Scientific (Invitrogen), Waltham, MA, USA	A11004	1:500
Anti-P53 antibody	Proteintech, Rosemont, IL, USA	60283-2-Ig	1:20,000
Anti-P21 antibody	Proteintech, Rosemont, IL, USA	10355-1-AP	1:2000
Anti-P27 antibody	Cell Signaling Technology, Danvers, MA, USA	2552	1:1000
Anti-GAPDH antibody	KangChen Bio-tech, Shanghai, China	KC-5G5	1:1000
HRP-conjugated secondary antibodies	KangChen Bio-tech, Shanghai, China	KC-MM-035	1:1000

## Data Availability

The raw data underlying this study are available from the corresponding authors upon reasonable request.

## Abbreviations

The following abbreviations are used in this manuscript:

HSC	Hematopoietic stem cell
HSPC	Hematopoietic stem and progenitor cell
scRNA-seq	Single-cell RNA sequencing
SD	Standard deviation
MOI	Multiplicity of infection
DPBS	Dulbecco’s phosphate-buffered saline
hCD45	Human cluster of differentiation 45
PCA	Principal component analysis
UMAP	Uniform manifold approximation and projection
HVG	Highly variable gene
GEO	Gene Expression Omnibus
HRP	Horseradish peroxidase
ECL	Enhanced chemiluminescence
SDS-PAGE	Sodium dodecyl sulfate-polyacrylamide gel electrophoresis
PVDF	Polyvinylidene fluoride
GAPDH	Glyceraldehyde-3-phosphate dehydrogenase
